# Conformational characterization of the mammalian-expressed SARS-CoV-2 recombinant receptor binding domain, a COVID-19 vaccine

**DOI:** 10.1186/s40659-023-00434-5

**Published:** 2023-05-08

**Authors:** Leina Moro-Pérez, Tammy Boggiano-Ayo, Sum Lai Lozada-Chang, Olga Lidia Fernández-Saiz, Kathya Rashida de la Luz, Jose Alberto Gómez-Pérez

**Affiliations:** Bioprocess R&D Department, Center of Molecular Immunology, 216 Street and 15 Avenue, Atabey, Playa, P.O. Box 16040, 11600 Havana, Cuba

**Keywords:** Characterization, Circular Dichroism, COVID-19 Vaccines, Fluorescence, RBD

## Abstract

The COVID-19 pandemic has caused a large number of diseases worldwide. There are few vaccines to constrain this disease and the value of them is high. In this sense, the antigens of the vaccine platform Soberana, the receptor binding domain from SARS-CoV-2 Spike protein, both the monomeric (mRBD) and dimeric (dRBD) forms, have been developed. This study encompassed several analyses by different techniques like circular dichroism (CD), fluorescence spectroscopy (FS) and Gel Filtration- High Performance Liquid ChLC of mRBD and dRBD. Monomer and dimer exhibited similar far-UV CD spectral characteristics with 54% of β-sheet content. Similar conformational features according to near-UV CD and FS studies were observed in both RBD. Stress stability studies by far-UV CD, FS, biological activity and GF-HPLC at 37 °C showed that mRBD is very stable. On the other hand, dRBD fluorescent emission showed a shift towards higher wavelengths as the incubation time increases, suggesting exposition of tryptophan residues, unlike what happens with mRBD. Biological activity outcome confirms these results. GF-HPLC profiles showed that in mRBD, the product of molecular stress are dimers and does not increase over time. However, dRBD showed dimer fragmentation as the main degradation species. This study reveals the usefulness of CD techniques for the analysis of degradation of RBD molecules as well as showed the difference in stability of both RBD molecules. Besides, our work provides useful insights into the production of a key protein used in diagnosis and therapeutics to fight COVID-19 pandemia.

## Introduction

In December 2019, a new type of coronavirus (SARS-CoV-2 or 2019-nCoV) causing a novel pneumonia named COVID-19 broke out in Wuhan, China. The virus was rapidly spreading cross the world and caused a great impact on health and economy [1].

Coronaviruses are enveloped non-segmented positive sense RNA viruses that have four open reading frames for structural proteins -Spike, Envelope, Membrane, and Nucleocapsid, from which Spike mediates the viral and cellular membrane fusion by binding mainly to the angiotensin-converting enzyme 2 (ACE2), a homologue of ACE [2].

One of more exposed and largest structural proteins of the Coronavirus is the Spike glycoprotein. The protein exists as a homotrimer where each monomer consists of 1,273 amino acid residues and is intertwined with each other. Each monomer has two domains, namely S1 and S2 [3]. Residues 319-591 from S1 correspond to the receptor binding domain from SARS-CoV-2 Spike protein (RBD), responsible for the interaction with ACE2. RBD binds with high affinity to the ACE2, located on the outer surface of the cell membrane, which acts as a SARS-CoV-2 receptor since it mediates the fusion of the virus to the cell membrane [4].

Art-state findings highlight the importance of the RBD domain of the spike protein in the SARS-CoV-2 vaccine design and provide a rationale for the development of a protective vaccine through the induction of antibodies against the RBD domain [5].

For this reason, the Center of Molecular Immunology (CIM) and Finlay Institute of Vaccine has developed the RBD molecules with the aim of using it in the vaccine against Covid-19 Soberana02 and Soberana Plus [6, 7].

**Fig. 1:**
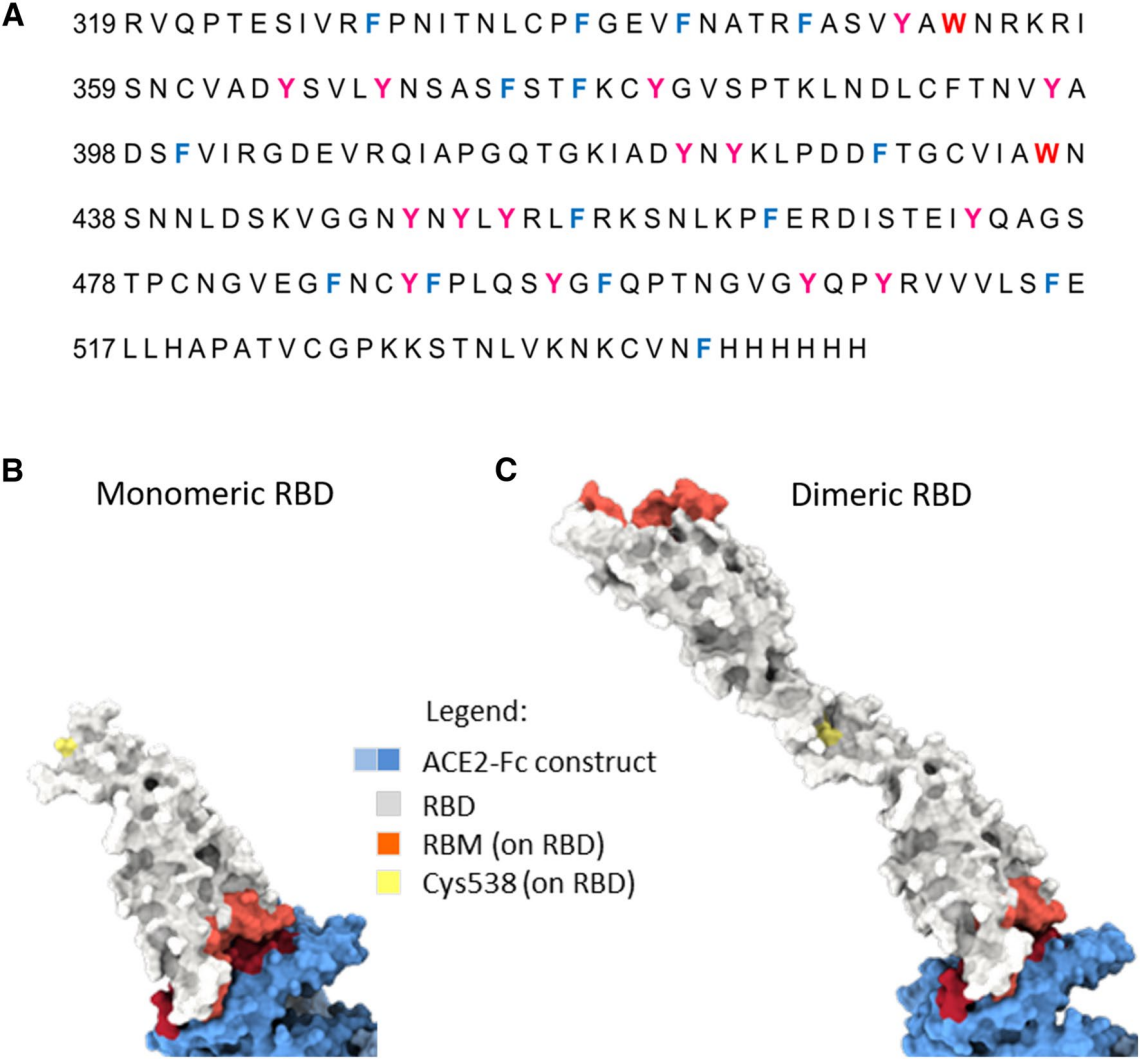
Structure of the RBD (319–541)-His6. **A** Amino acid Sequence by MALDI of SARS-CoV-2 RBD. Proteolytic degradation by Trypsin of RBD. Trp (red), Tyr (pink) and Phe (blue). **B** Tridimensional structure of monomeric RBD. **C** Tridimensional structure of the dimeric RBD. 3D structures of RBD proteins were performed using the PyMOL Molecular Graphics System from PDBs 6M0J. In pink: the Receptor-binding motive (RBM), red: residues in contact with the ACE2, blue: glycosylation motives. S–S: disulfide bond between the Cys528 of each monomeric RBD

Variants of RBD with different lengths have been expressed in disparate cell hosts [2, 8]. Our lab developed a recombinant RBD protein transfected in CHO cell line in order to preserve the mammalian glycosylation structure [6]. This recombinant RBD has 229 amino acids: from R319 to F541 plus a His-tag with six histidine in the N-terminal of the protein (Fig. 1A).

A total of nine cysteine residues are found in the RBD, eight of which form four pairs of disulfide bonds that are resolved in the final model. Among these four pairs, three are in the core (Cys336–Cys361, Cys379–Cys432 and Cys391–Cys525), which help to stabilize the β-sheet structure; the remaining pair (Cys480–Cys488) connects the loops. The Cys528, which in the original Spike S protein is paired with the Cys590, remains unpaired in the RBD(319–541) molecule. The ninth unpaired cysteine could be a challenge for the expression and stability of the protein, but plays an important role for the Soberanas vaccines (Fig. 1B, C). Cys528 allows the dimerization of the protein as well as the chemical conjugation with the immunogenic carrier Tetanic Toxoid (TT) [6, 7]. With a molecular weight (MW) of 24,183.18 g/mol for monomeric RBD (mRBD) and 48,366.36 g/mol for dimeric RBD (dRBD), both molecules are the Biological Active Ingredient for the Soberanas vaccines.

RBD has two potential N-glycosylation (N331 and N343) and two O-glycosylation sites (Thr323 and Ser325). The addition of glycan moieties might have a relevant role in the “in vivo*”* protein folding process, in the dynamics, stability and solvent accessibility of RBD [2]. RBD is not a globular protein domain; it has a central twisted antiparallel beta-sheet formed by five strands decorated with secondary structure elements (short helices and strands) and loops [2, 9].

As part of the physical–chemical knowledge that is required by regulation to request the Clinical Trial Authorization of any candidate [10], it is necessary to carry out a study of conformational and structural characterization of the active pharmaceutical ingredient, in this case, monomeric and dimeric RBD.

Circular Dichroism (CD) and Fluorescence Spectroscopy (FS) are among the techniques of characterization used in the biopharmaceutical industry to study the effects that manufacturing, formulation and storage procedures have on the conformation and stability of proteins [11].

CD is a nondestructive spectroscopic technique used to obtain low-resolution structural information on biological macromolecules such as proteins or nucleic acids [12]. In the far UV region (240–180 nm), which corresponds to peptide bond absorption, the CD spectrum can be analyzed to give the content of regular secondary structural features such as α-helix and β-sheet [13]. The CD spectrum in the near UV region (320–260 nm) reflects the environments of the aromatic amino acid side chains and thus gives information about the tertiary structure of the protein [14]. Tryptophan fluorescence is very sensitive to protein conformational changes and provides valuable information about changes in protein secondary and tertiary structure [12].

In this case, we present a conformational characterization and stability by circular dichroism (CD), fluorescence spectroscopy (FS), biological activity and Gel Filtration- High Performance Liquid Chromatography (GF-HPLC). Our work evidences the differences in the stability of the monomer and dimer of RBD. Furthermore, we show the FS as a powerful tool for the follow-up stability of the RBD (319–541)-His6 despite being described as a low-resolution structural information technique.

## Results

### Physicochemical characterization

**Fig. 2:**
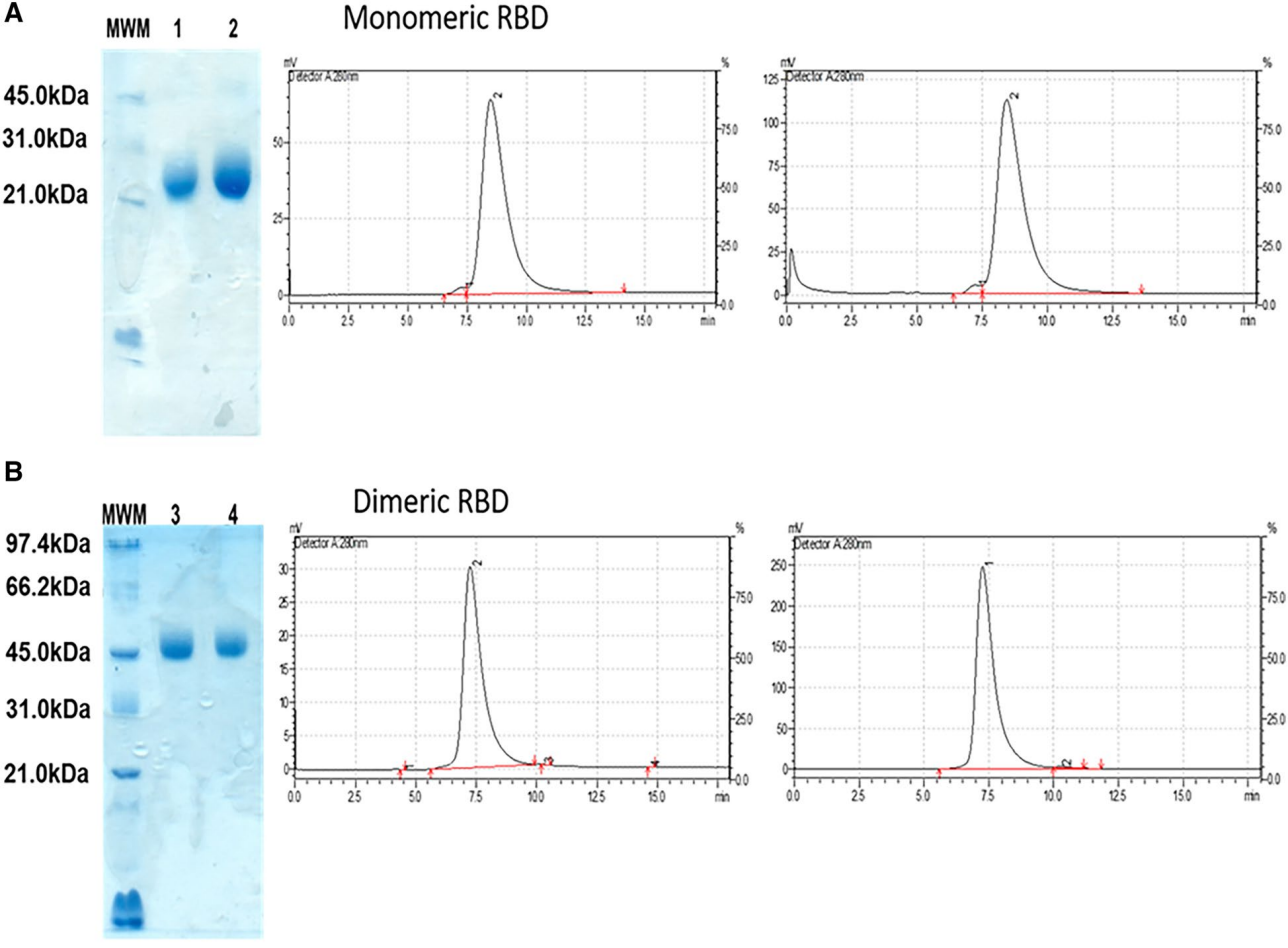
SDS-PAGE non-reduced and GF-HPLC for monomeric RDB and dimeric RBD.** A** Electrophoretic profile by SDS-PAGE and chromatographic GF-HPLC profile of the monomeric RBD samples: mRBD Lot 1 (lane 1) and mRBD Lot 2 (lane 2). **B** Electrophoretic profile by SDS-PAGE and chromatographic GF-HPLC profile of the dimeric RBD samples: dRBD Lot 1 (lane 3) and dRBD Lot 2 (lane 4). Legend. MWM, Molecular Weight size Marker. About 5 µg of monomeric or dimeric form were applied to a 7% Tris/glycine-SDS–PAGE gel with non-reducing conditions and then stained with Coomassie brilliant blue

The results obtained from the analysis of the SDS-PAGE and the GF-HPLC chromatograms (Fig. 2) indicated that the monomer and dimer to be characterized for CD and FL had a high purity. On the SDS-PAGE, a band was observed at approximately 24 kDa (Fig. 2A) corresponding to the monomeric form of the RBD and another band at 48 kDa (Fig. 2B) corresponding to the dimeric form of the RBD. In the GF-HPLC a single elution peak was also obtained for all the evaluated samples.

### Determination of RBD secondary structure by far-UV CD

**Fig. 3:**
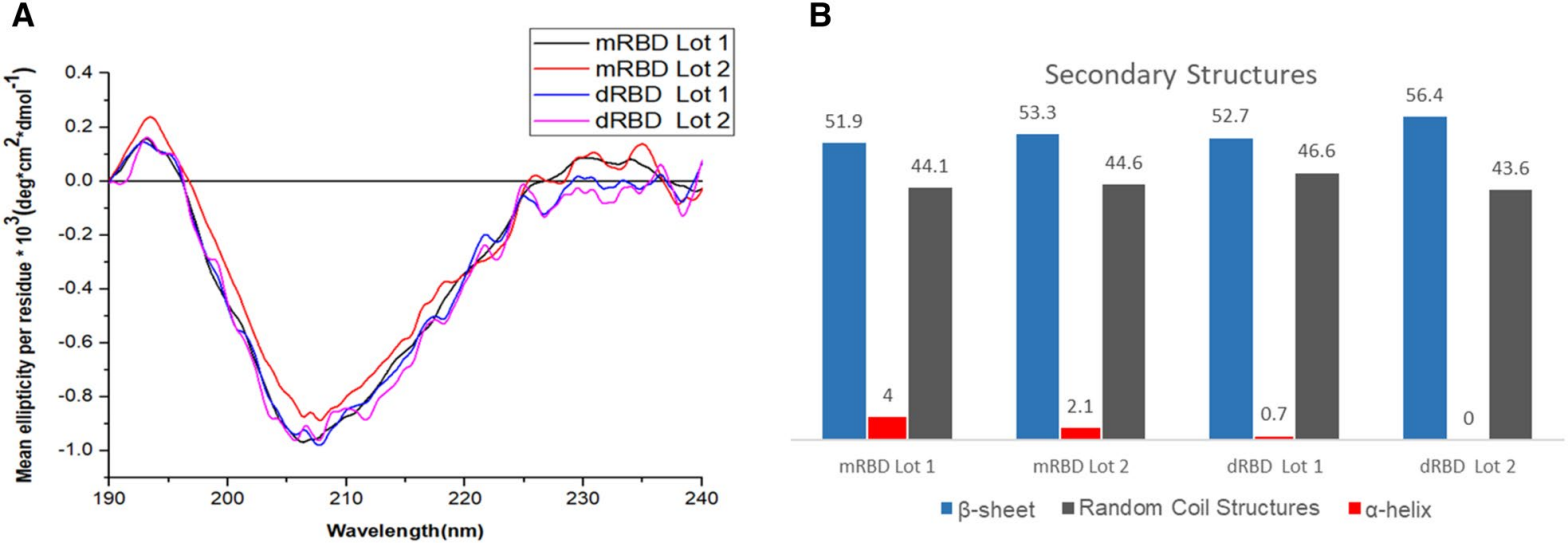
Far-CD UV spectra and BeStSel results of the mRBD and dRBD.** A** Lots of the RBD are shown: mRBD Lot 1 (black), mRBD Lot 2 (red), dRBD Lot 1 (blue) and dRBD Lot 2 (pink). Each is the average of 5 accumulations in the range of 190 to 240 nm, with 4.13 µmol/l and 2.07 µmol/l of dRBD in water, in quartz cuvettes of 0.1 cm of light path. The average residual ellipticity values (deg.cm^2^.dmol^−1^) for each wavelength were obtained on a Jasco J-1500 spectropolarimeter (Jasco Corporation, Japan) at 25 °C; 0.1 nm of intervals and constant speed of 20 nm/min with 8 s of response time. The spectra were filtered with the fast Fourier transform (FFT) in a 20-point window to smooth out the curve in the Origin-v15.0 program (Origin Lab Corporation, USA). **B** Secondary structure content (%) of mRBD and dRBD are shown: β-sheet (blue), random coil structures (gray) and α-helix (red). The content of secondary structures was determined using the BeStSel internet server and the SELCON III algorithm. The deconvolution analysis of the CD spectra obtained in the far-UV shows similar percentages of α-helix, β-sheet and random coil structures for all lots

First, to avoid high signal/noise ratio in far-UV CD assay, the mRBD and dRBD were desalted by change of buffer to milliQ water [15] with molecular exclusion chromatography. Next, the secondary structure of RBD (in milliQ water) was determined from the far-UV CD analysis of the spectra (190 to 240 nm). Figure 3A shows similar far-UV CD spectra of the mRBD and dRBD, suggesting a similar secondary structure. The spectra showed the traits of proteins with high β-sheet content, which is characterized by a positive band around 193 nm and a negative band at approximately 206 nm [11]. In addition, a maximum at 230 nm was observed, suggesting the contribution of aromatic residues to the spectra [2].

The content of secondary structures was determined using the BeStSel Internet server and the SELCON3 algorithm. The analysis of the Far-UV CD spectra deconvolution showed similar percentages of α-helices, β-sheets and disordered structures for mRBD and dRBD (Fig. 3B). A predominant occurrence of β-sheets structure was also confirmed.

### Tertiary structure analysis of the mRBD and dRBD by near-UV CD

**Fig. 4:**
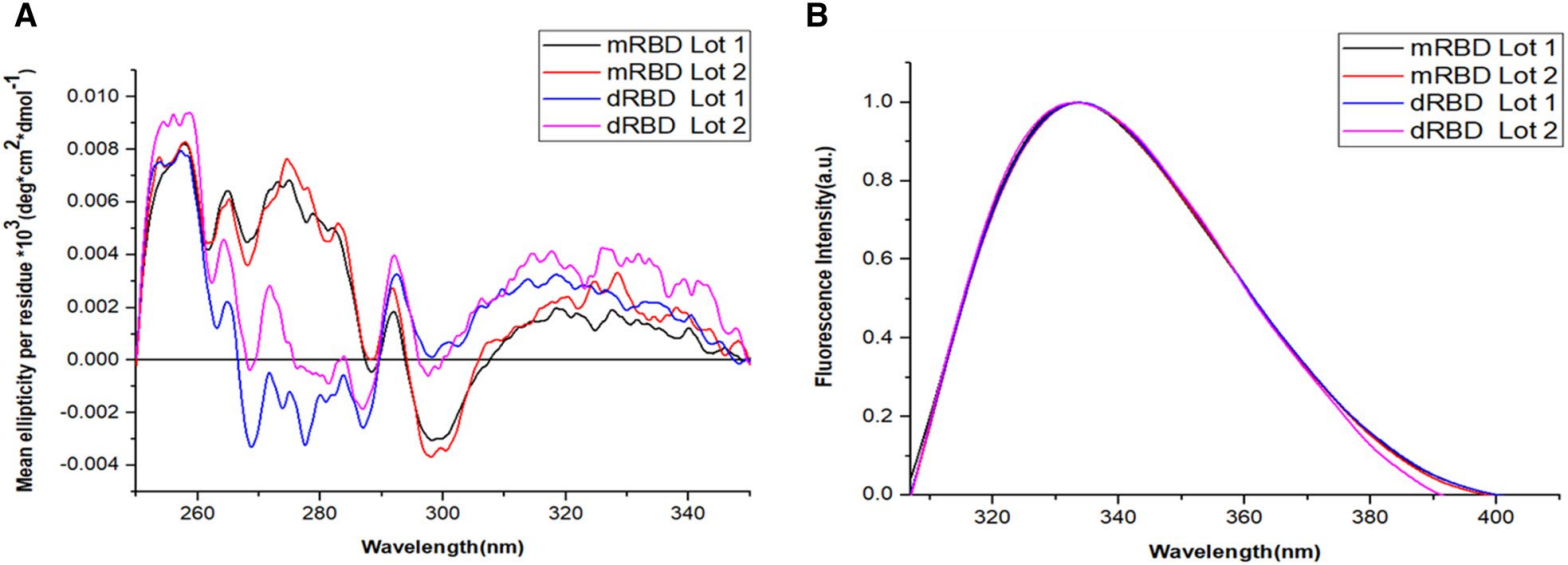
Near-UV CD and FS spectra of the mRBD and dRBD. Lots of the RBD are shown: mRBD Lot 1 (black), mRBD Lot 2 (red), dRBD Lot 1 (blue) and dRBD Lot 2 (pink). **A** Near-UV CD spectra, where each spectrum is the average of 3 scans in the 250–350 nm range, with 41.35 µmol/l of mRBD and 20.67 µmol/l of dRBD, in quartz cuvettes of 1 cm of light path. The mean residual ellipticity readings (deg.cm^2^.dmol^−1^) for each wavelength were performed on a Jasco J-1500 spectropolarimeter (Jasco Corporation, Japan) at 25 °C; 0.1 nm of intervals and constant speed of 10 nm/min with 16 s of response time. The spectra were filtered with the FFT in a 20-point window to smooth out the curve in the Origin-v15.0 program (Origin Lab Corporation, USA). **B** FS spectra**,** where each spectrum represents the fluorescence emission intensity (a.u) for each wavelength (nm) and is the product of a single scan in the 307–410 nm range. Selective Trp excitation was performed at 295 nm, with 5 nm slits for excitation and emission, respectively. Protein concentration 4.13 µmol/l of mRBD and 2.07 µmol/l of dRBD. Spectra were acquired using a Jasco J-1500 spectropolarimeter (Jasco Corporation, Japan), in quartz cuvettes with a 1 cm light path, at 25 °C

The information on the global conformation of mRBD and dRBD was analyzed by near-UV CD (250 to 350 nm). As shown in Fig. 4A**,** positive absorption bands corresponding to Phe were observed, approximately at 258 nm and 256 nm for mRBD and dRBD, respectively. Furthermore, between 270 and 285 nm there were positive and negative bands (only for dRBD) corresponding to Tyr. An absorption maximum of Trp was also observed in the spectra at approximately 292 nm. All evaluated lots showed a three-dimensional folding of the RBD, with the presence of the absorption bands characteristic of aromatic amino acids as reported before [11, 16].

### Conformational features of the mRBD and dRBD by Trp intrinsic fluorescence

Conformational characteristics of mRBD and dRBD were determined by fluorescence emission in the wavelength range from 307 to 410 nm, following selective excitation of Trp at 295 nm (Fig. 4B). All the spectra of evaluated batches were very similar respective to the maximum emission wavelength (emission maxima: 333 – 334 nm). These results indicate that there is a similarity in the conformational environment of the Trp.

### RBD stress stability

**Fig. 5:**
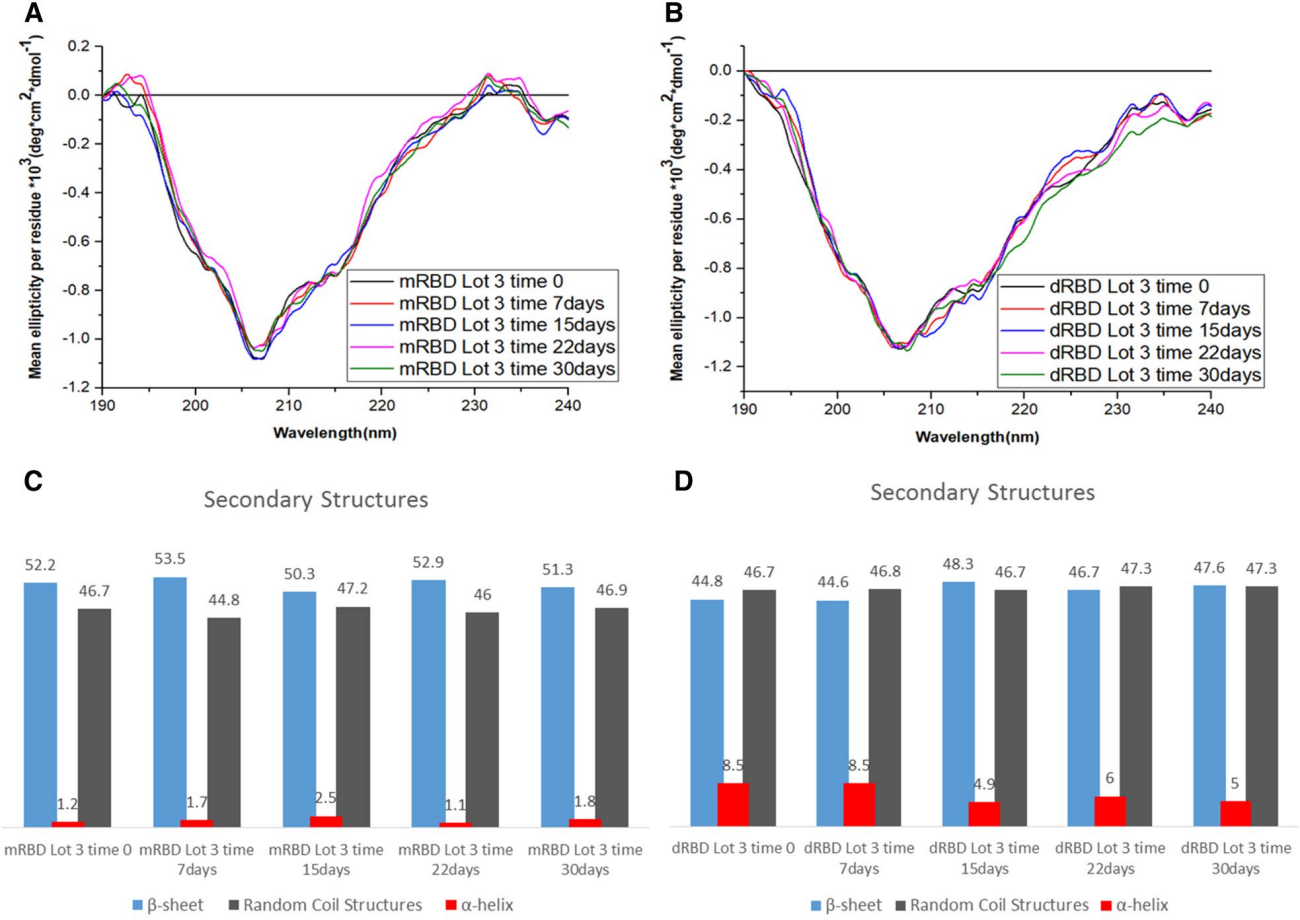
Stress stability at 37 °C of mRBD and dRBD measured by Far-UV CD and BeStSel **A** Lots of the mRBD are shown: mRBD Lot 3 time 0 (black), mRBD Lot 3 time 7 days (red), mRBD Lot 3 time 15 days (blue), mRBD Lot 3 time 22 days (pink) and mRBD Lot 3 time 30 days (green). **B** Lots of the mRBD are shown: mRBD Lot 3 time 0 (black), mRBD Lot 3 time 7 days (red), mRBD Lot 3 time 15 days (blue), mRBD Lot 3 time 22 days (pink) and mRBD Lot 3 time 30 days (green). Each is the average of 5 accumulations in the range of 190 to 240 nm, with 4.13 µmol/l of mRBD and 2.07 µmol/l of dRBD in water and PBS, respectively, in quartz cuvettes of 0.1 cm of light path. The average residual ellipticity values (deg.cm^2^.dmol^−1^) for each wavelength were obtained on a Jasco J-1500 spectropolarimeter (Jasco Corporation, Japan) at 25 °C; 0.1 nm of intervals and constant speed of 20 nm/min with 8 s of response time. The spectra were filtered with the fast Fourier transform (FFT) in a 20-point window to smooth out the curve in the Origin-v15.0 program (Origin Lab Corporation, USA). **C**, **D** Secondary structure content (%) of mRBD and dRBD are shown: β-sheet (blue), random coil structures (gray) and α-helix (red). The content of secondary structures was determined using the BeStSel internet server and the SELCON III algorithm. The deconvolution analysis of the CD spectra obtained in the far-UV shows similar percentages of α-helix, β-sheet and random coil structures for all lots

In order to check the degradation species and compare the stability of monomer and dimer of RBD, samples of both molecules were kept at 37 °C for 30 days. Analysis of structure, purity and ACE-2 binding of samples were done on 0, 7, 15, 22 and 30 days**.** The secondary structure of RBD (in milliQ water) was determined from the far-UV CD analysis of the (190 to 240 nm) spectra. The far-UV CD spectra of the mRBD and dRBD were similar as illustrated in Fig. 5A, B. This result suggests a similar secondary structure. A positive peak at 193 nm and a negative peak at 206 were obtained for mRBD. A positive peak was also observed at 230 nm, which points out the contribution of Cys residues (9 in this case). In contrast, for dRBD only one negative peak was observed at 206 nm.

Next, we evaluated the percentages of secondary structures for mRBD and for dRBD. For the different incubation times there were no differences in the structure proportion (Fig. 5C, D).

**Fig. 6:**
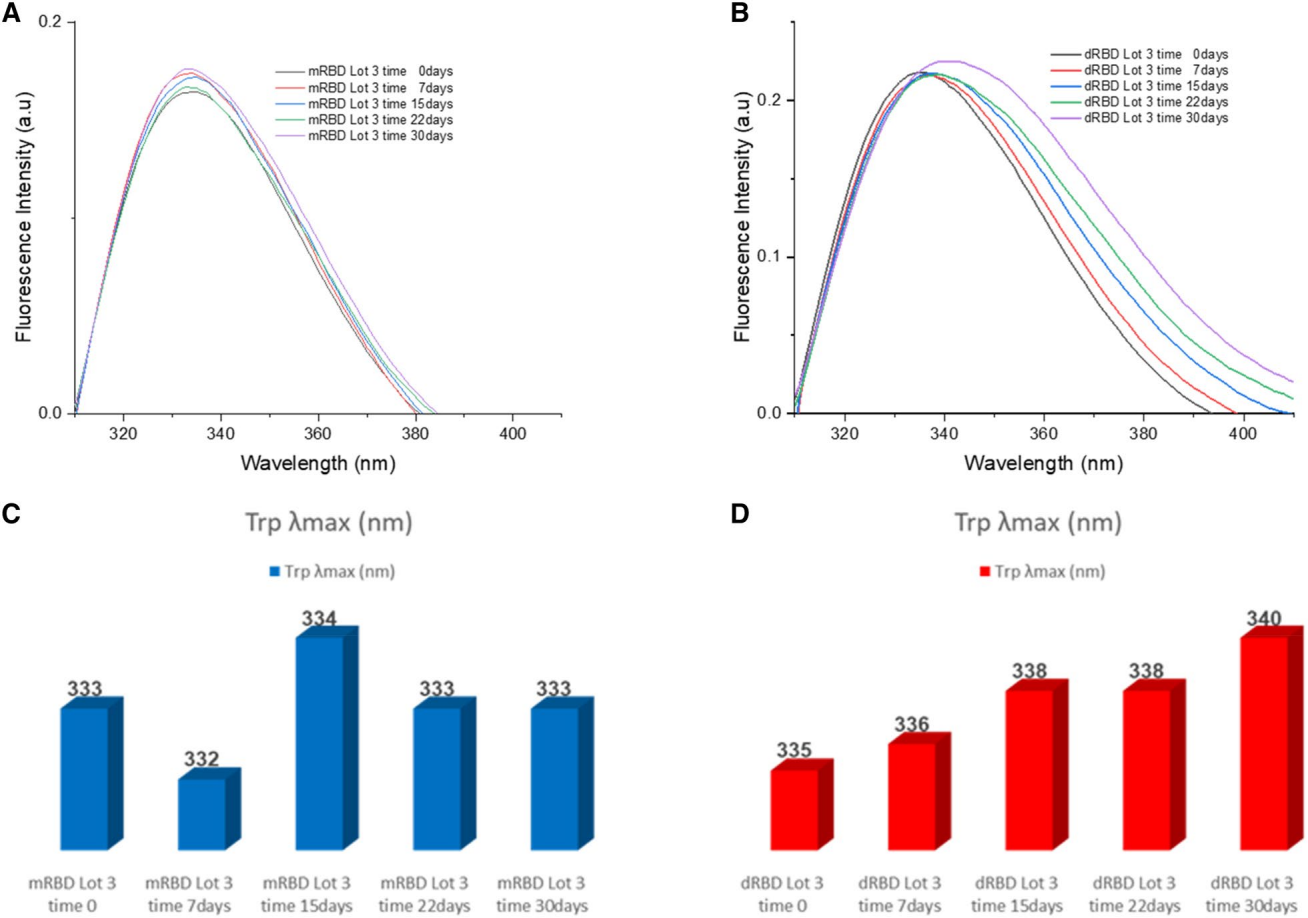
Stress stability at 37 °C of mRBD and dRBD measured by FS. **A** Lots of the mRBD are shown: mRBD Lot 3 time 0 (black), mRBD Lot 3 time 7 days (red), mRBD Lot 3 time 15 days (blue), mRBD Lot 3 time 22 days (pink) and mRBD Lot 3 time 30 days (green). **B** Lots of the mRBD are shown: mRBD Lot 3 time 0 (black), mRBD Lot 3 time 7 days (red), mRBD Lot 3 time 15 days (blue), mRBD Lot 3 time 22 days (pink) and mRBD Lot 3 time 30 days (green). FS spectra**,** where each spectrum represents the fluorescence emission intensity (a.u.) for each wavelength (nm) and is the product of a single scan in the 307–410 nm range. Selective Trp excitation was performed at 295 nm, with 5 nm slits for excitation and emission, respectively. Protein concentration 4.13 µmol/l of mRBD and 2.07 µmol/l of dRBD. Spectra were acquired using a Jasco J-1500 spectropolarimeter (Jasco Corporation, Japan), in quartz cuvettes with a 1 cm light path, at 25 °C. **C**, **D** Bar graph of the Trp λmax for the mRBD and dRBD. Trp λmax of the RBD are shown: mRBD (blue) and dRBD (red)

Conformational characteristics of mRBD and dRBD were determined by fluorescence emission in the wavelength range from 307 to 410 nm, following selective excitation of Trp at 295 nm (Fig. 6). All the spectra of the different incubation times of the mRBD batch were very similar (emission maxima: 332 – 334 nm) (Fig. 6A and C). However, it was observed that for the dRBD batch, the Trp emission maxima shift towards higher wavelengths as the incubation time increases (Fig. 6B and D). On the other hand, the maximum intensity of the FS showed slight differences between samples incubated at 37 °C at different times.

**Fig. 7:**
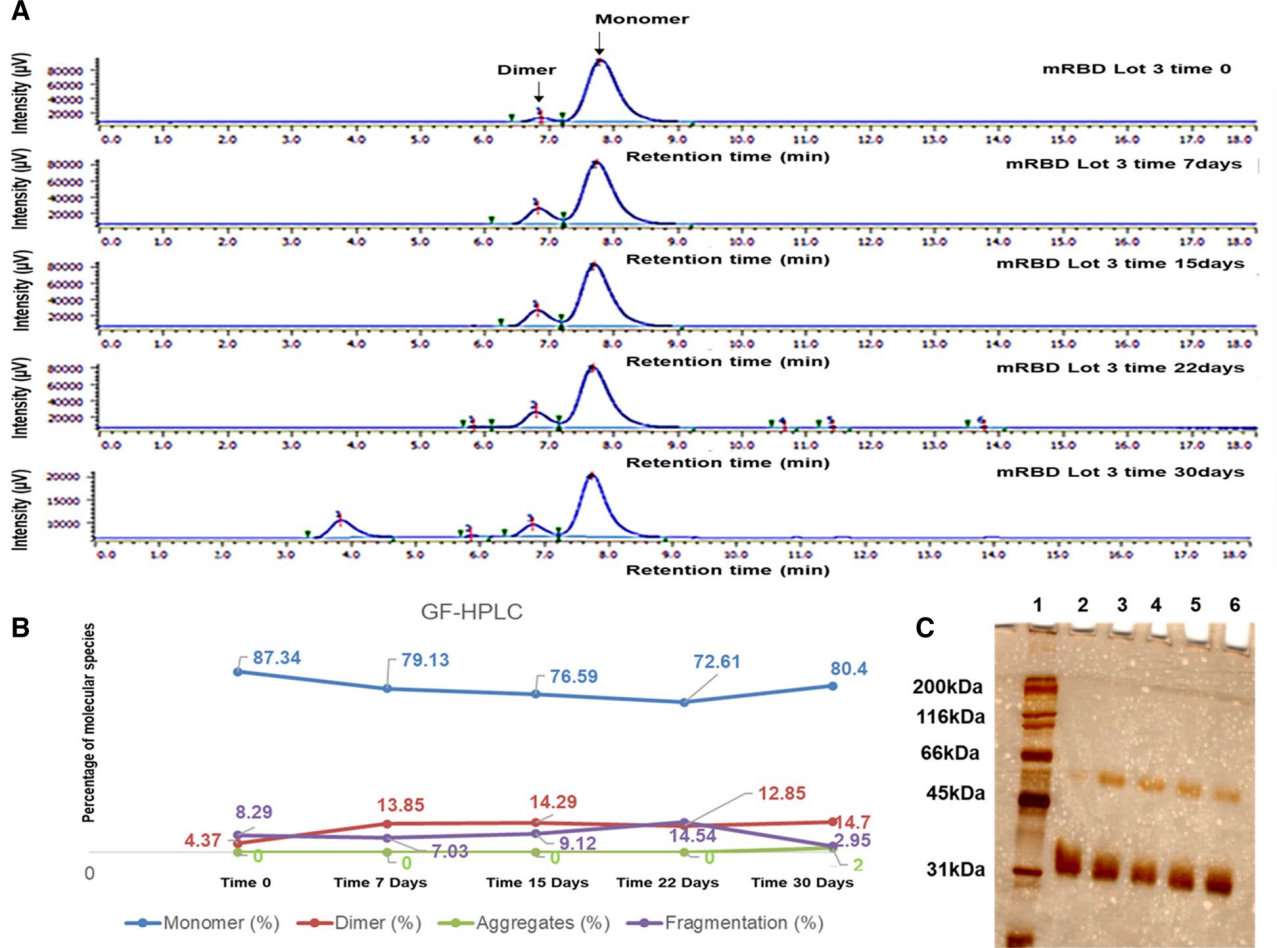
Purity of monomeric RBD during a stress stability at 37 °C measured by GF-HPLC and SDS PAGE.** A** mRBD chromatograms by GF-HPLC during storage at 37 °C. **B** XY graph of the percentages of the species calculated from the area under the curve of the peaks corresponding to the mRBD. Lots of the mRBD are shown: **Point 1:** mRBD Lot 3 time 0, **Point 2:** mRBD Lot 3 time 7 days, **Point 3:** mRBD Lot 3 time 15 days, **Point 4:** mRBD Lot 3 time 22 days and **Point 5:** mRBD Lot 3 time 30 days. Percentage of the mRBD are shown: monomer (blue), dimer (pink), aggregates (red) and fragmentation (green). **C** Electrophoretic profile by SDS-PAGE of the mRBD at different concentrations of stress (**Point 1:** MWM, Molecular Weight size Marker, **Point 2:** mRBD Lot 3 time 0, **Point 3:** mRBD Lot 3 time 7 days, **Point 4:** mRBD Lot 3 time 15 days, **Point 5:** mRBD Lot 3 time 22 days and **Point 6:** mRBD Lot 3 time 30 days)

To complement the results obtained by far CD-UV and FS, we compared the chromatographic and electrophoretic profiles of mRBD and dRBD batches by GF-HPLC and SDS-PAGE, respectively. As Fig. 7 show, from day 7 to 30, there were no changes in the percent of monomers and dimers (Fig. 7B). Despite the light dimerization at the beginning of the experiment, between days 0 and 7, mRBD seems to be stable.

**Fig. 8:**
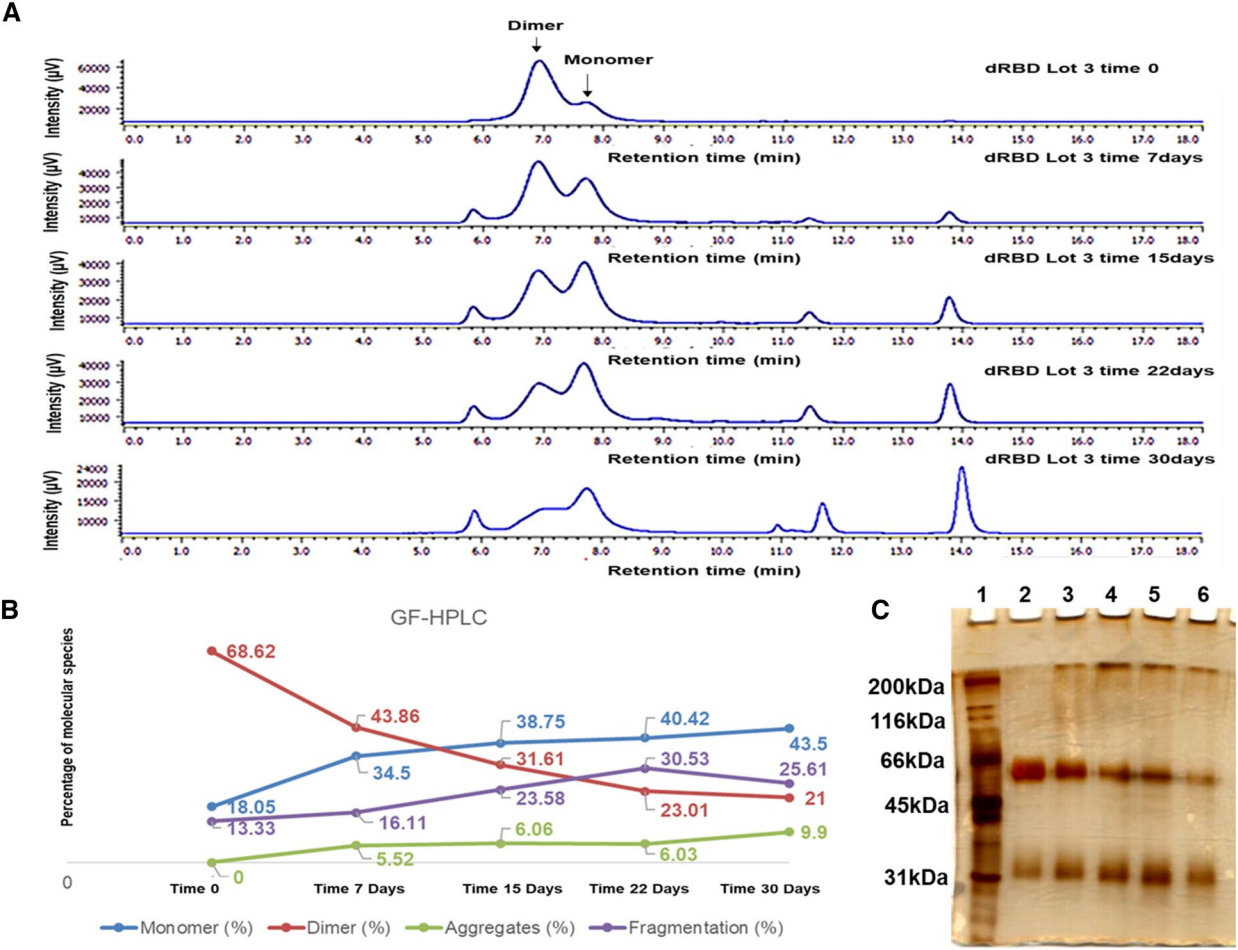
Purity of dimeric RBD during a stress stability at 37 °C measured by GF-HPLC and SDS PAGE. **A** dRBD chromatograms by GF-HPLC during storage at 37 °C. **B** XY graph of the percentages of the species calculated from the area under the curve of the peaks corresponding to the dRBD. Lots of the dRBD are shown: **Point 1:** dBD Lot 3 time 0, **Point 2:** dRBD Lot 3 time 7 days, **Point 3:** dRBD Lot 3 time 15 days, **Point 4:** dRBD Lot 3 time 22 days and **Point 5:** dRBD Lot 3 time 30 days. Percentage of the dRBD are shown: monomer (blue), dimer (red), aggregates (green) and fragmentation (purple). **C** Electrophoretic profile by SDS-PAGE of the mRBD at different concentrations of stress (**Point 1:** MWM, Molecular Weight size Marker, **Point 2:** dRBD Lot 3 time 0, **Point 3:** mRBD Lot 3 time 7 days, **Point 4:** mRBD Lot 3 time 15 days, **Point 5:** mRBD Lot 3 time 22 days and **Point 6:** mRBD Lot 3 time 30 days)

Besides, dRBD decreased the proportion of dimers from 68 to 21%, increasing at the same time the percent of monomers up to 43% and fragment up to 25% (Fig. 8A, B). The aggregation increase was not remarkable for dRBD.

### Influence of thermal stress in biological activity

**Fig. 9:**
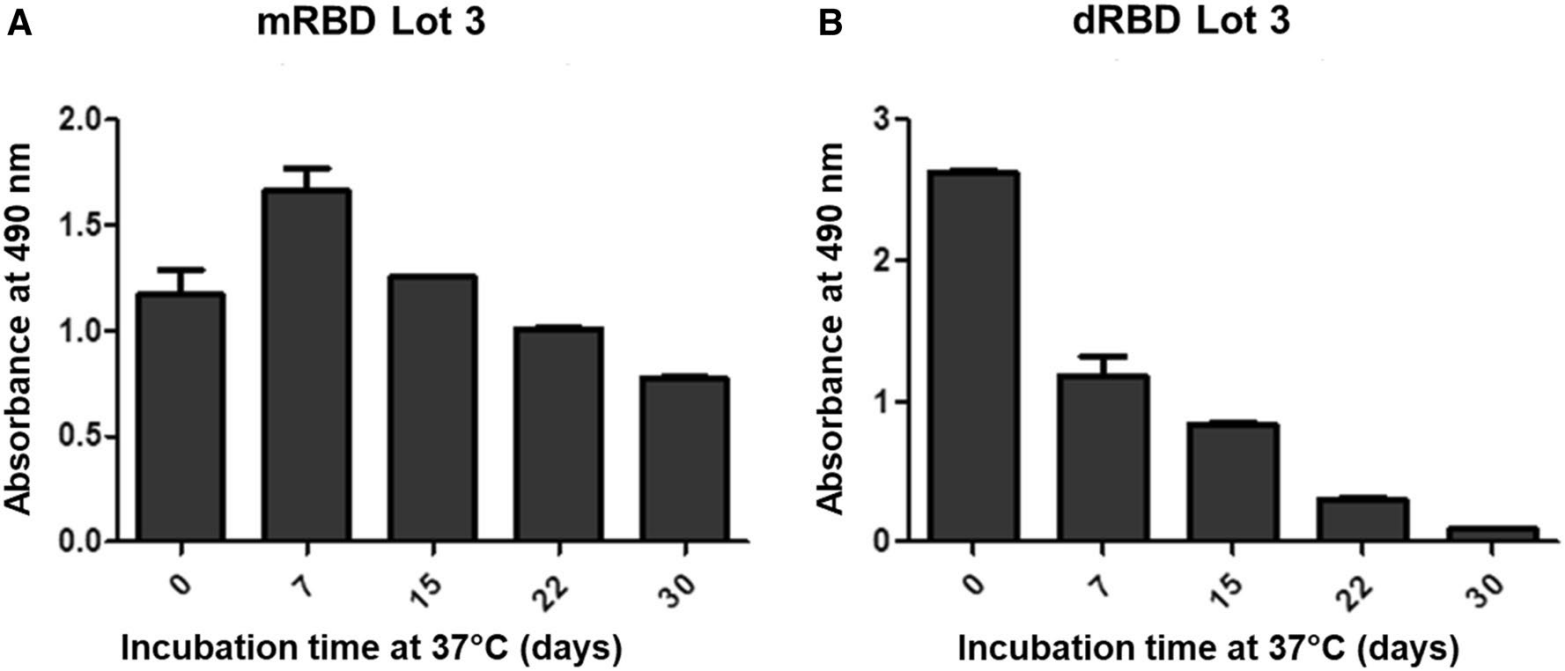
Behavior of the ACE2 binding activity of samples mRBD and dRBD during a stress stability at 37 °C. Biological activity measured as the binding of the monomeric and dimeric RBD protein to the ACE2 receptor by a sandwich ELISA. **A** Bar graph of the absorbance at 490 nm of the monomeric RBD (mRBD) samples keeping at 37 °C at different times. **B** Bar graph of the absorbance at 490 nm of the dimeric RBD (dRBD) samples keeping at 37 °C at different times

During the stress study at 37 °C we set out to evaluate the biological activity through the ability of the mRBD and dRBD to bind the ACE2 receptor, by ELISA. There were differences in the sensitivity of biological activity to stress conditions over time between mRBD and dRBD (Fig. 9). As seen in Fig. 9, the binding of mRBD to ACE2 showed a slight increase after seven days and then remained stable. However, the dimeric species was less stable in its behavior, observing a substantial reduction in binding to ACE2 on day 30 of the study.

## Discussion

In the present work, it is shown a conformational characterization study and stability of a novel RBD by means of CD, Trp intrinsic fluorescence, electrophoresis and GF-HPLC. The analysis of the secondary structure by far-UV CD showed typical spectral characteristics of β-sheets for mRBD and dRBD, similar to those previously reported for RBD from SARS-CoV-2 Spike protein expressed in the well-established HEK-293 T mammalian cell system [2]. Therefore, the structures of the attachment proteins of CoVs, influenza, measles, HIV and Ebola viruses display higher proportions of β-strands, whereas α-helix is under-represented. Among non-regular secondary structures, the random coil is the most over-represented assignment [17]. This result was corroborated when the percentage of secondary structures for both RBD was compared using the BeStSel tool (51.9 to 56.4% of β-sheets). Besides, structural studies showed that the SARS-CoV-2 RBD has a twisted five-stranded antiparallel β sheet, with short connecting helices and loops that form the core [9]. Similar results to those reported in the analysis of the secondary structure by far-UV CD were obtained in the near-UV CD, where the spectra showed the appearance of absorption bands corresponding to the aromatic residues [11].

The near-UV CD spectra for the mRBD and dRBD batches were very similar in the position of the aromatic amino acid absorption bands with differences in signal intensity. The greatest variation observed among the evaluated samples was in the Tyr absorption region. There are several factors that influence the intensity of the bands assigned to aromatic residues: the rigidity of the protein; the intensity being lower the higher the mobility; the interactions between aromatic groups, which become very important at distances less than 1 nm; and the number of aromatic groups [18]. The near-UV CD spectra of the mRBD batches show lower intensity values in the Phe and Trp regions with respect to the dRBD batches. These differences, following the previous reasoning, can be attributed to the fact that the mRBD has less rigidity since being in monomeric form it has greater freedom of movement.

These results illustrate that these proteins showed similar secondary structure content, but the conformations of the aromatic side chains may be different. This characteristic can be addressed since the near-UV CD spectrum being sensitive to the different orientation of the aromatic side chains [19].

The Trp emission maximum, obtained by excitation at 295 nm, was very similar for the mRBD and dRBD samples evaluated (332–334 nm). This result is in correspondence with that reported in the state of the art for proteins [20] and for the RBD from SARS-CoV-2 Spike protein expressed in HEK-293T cell line [2]. This outcome suggests that these amino acids are found in an apolar environment [21], presumably in the vicinity of apolar amino acid residues, or buried within the globular structure of the molecule, since Trp is very sensitive to the polarity of the medium [20].

The knowledge of the main degradation species of RBD products and identifying sensitive and specific analytical methods that allow detecting the possible changes that it may present during its storage is of paramount importance for product development. Temperature is a very significant variable for proteins because proteins respond differently in high and low temperature conditions. Many proteins have high thermal stability while others can unfold or even denature at high temperatures [3]. Klausberger et. al. reported at 37 °C the induction of protein unfolding for SARS Cov-2 RBD expressed in HEK-293 T cell line [22]. In our case, 37 °C was chosen as the stress temperature to have a mild condition, less stressful than the higher temperatures usually used for this kind of study; therefore, differences of stability between monomers and dimers can be established.

In the stress stability study of RBD, the far-UV CD spectra for the different incubation times for mRBD and for dRBD were very similar. In the case of mRBD, spectra similar to those obtained in the characterization study previously carried out for two batches of mRBD were observed (Fig. 3), which in turn coincides with the art states for this type of molecule [2, 9].

The emission spectra of Trp by fluorescence for the different incubation times of the mRBD batch were very similar (emission maxima: 332–334 nm). However, it was observed that for the dRBD batch, the Trp emission maxima shift towards higher wavelengths as the incubation time increases (dRBD time 0: 335 nm and dRBD time 30 days: 340 nm) (Fig. 6). Additionally, differences in the maximum fluorescent intensity appear. The explanation for these differences observed would be that the indole group is highly sensitive to the polarity of the solvent, so that its emission can change at wavelengths close to those of the blue color if the group is buried within a native protein. Its emission can change to longer wavelengths when the protein is deployed and this group is exposed to the solvent [20]. A possible explanation for the slight variations in the intensity of the spectra of mRBD and dRBD could be related to the quenching of the fluorescence of those groups as their conformational environment changes.

On the other hand, the evaluation carried out in GF-HPLC and SDS-PAGE on the mRBD batch, despite the slight augment in the dimeric species after 7 days of storage at 37 °C, the molecule remaining similar until day 30 (Fig. 7). We can affirm that in mRBD, the product of molecular stress is dimer. Its content rose up to 14% in the first week of the study and did not increase over time.

In contrast, the chromatographic profiles obtained in GF-HPLC and SDS-PAGE for the dRBD batch showed a decrease in the dimeric species and a rise in the monomeric species, indicative of the occurrence of reduction of the intermolecular disulfide bridge (Fig. 8). During the evaluated period, the percentage of fragmentation increases proportionally to the exposure time at a temperature of 37 °C. We can affirm that dRBD has a complex degradation pattern where both dimer fragmentation products that give rise to molecules of the size of monomer (40%) and smaller fragments that represent 30% arose during the exposure time.

The stability of dimers has been compared with that of monomers in a fortuitous way in the literature. Trivedi et. al. described the higher instability of the dimeric protein EC1 compared with its monomeric counterpart [23].

In the state of the art, testing of the thermal stability of the RBD mutants was performed with differential scanning calorimetry (DSC). Interestingly, the midpoint transition temperature (TM) of the size-exclusion chromatography (SEC)-purified dimeric wild-type RBD (49.6 °C) was considerably lower than that of monomeric RBD (53.0 °C), indicating partial misfolding and was similar as determined in our work [22]. The possible cause of this behavior could be the presence of a mixture of conformational state in the dRBD due to scrambled disulfide bridges. The scrambled disulfide bonds tend to be more unstable structures prone to degradation.

The GF-HPLC and FL results obtained in the stress stability study were corroborated by the biological activity assay. dRBD showed a decrease of the ACE-2 binding along the incubation time while mRBD presented a slight increase in binding to ACE2 receptor at seven days of incubation in thermal stress (Fig. 9), which could be explained by the increase in percentage of dimers observed in GF-HPLC. It is also known from previous experiments that the dimeric species are more reactive than the monomeric one [24]. It is advisable to store the dimer at a lower temperature than the monomer. Stability study at the final storage temperature should be done to evaluate the holding time of the biological active ingredient until the vaccine formulation.

## Conclusions

Conformational characterization and stress stability studies reveal in the first place that the dimeric RBD was less stable than the monomeric one and, in the second place, that the techniques like Trp fluorescence are suitable to follow the stability of both RBD molecules. Both biological active ingredients could be used as a component of a potential vaccine antiCOVID-19.

## Materials and methods

### Materials

RBD (Spike residues from 319 to 541) was produced in transfected in Chinese Hamster Ovary cells (CHO) expression system with C-terminal (525 to 541) fused to a His6 tag for purification (Fig. 1). Purification schedule started with IMAC capture and finished with a size exclusion step for obtaining the monomeric and dimeric RBD molecules. It have nine cysteine residues (eight of them forming disulfide bonds). Free or unpaired Cys538 enables the monomeric sequence to dimerize forming a new disulfide bridge through the Cys538 of two monomers.

Three batches of the active product ingredient (API) of the mRBD and dRBD were used, where the formulation of the API is 20 mmol/L phosphate-buffered saline (PBS) at pH 7.4.

### Molecular exclusion chromatography

The SARS-CoV-2 RBDs were desalted by a change of buffer with exclusion chromatography using a Nap-5 column equilibrated with 10 ml of milliQ water. Then, 100 μl of each sample were injected, equilibrated with 400 μl of milliQ water and fractions of 500 μl were collected.

### Determination of protein concentration

Protein concentration in PBS was determined by bicinchoninic acid (BCA) Protein Assay Kit (ThermoScientific, USA). The point calibration curve was performed in duplicate inside the concentration range from 0.02 mg/ml to 0.18 mg/ml. The absorbance of the samples was measured on a spectrophotometer at 620 nm.

The mass concentration was converted to amount of substance concentration (mol/L) using the MW value corresponding to a Sars-Cov-2 mRBD of 24,183.18 g/mol and a Sars-Cov-2 dRBD of 48,366.36 g/mol.

### SDS-PAGE

RBD samples (5 µg) in monomeric or dimeric form were separated in a 7% Tris/glycine-SDS-PAGE gel under non-reducing conditions. The electrophoresed gels were stained using Coomassie Brilliant Blue R250, destained with a mixture of methanol and acetic acid or with silver as described elsewhere [25] and processed with a densitometer equipped with Image Lab software (Biorad, USA).

### Analytical GF-HPLC

J-PU4086 pumps and J-UV4070 detector (Jasco, Japan) were operated to determine the size and purity of purified RBD molecular species. Five micrograms of the RBD were injected into a TSK2000 (7.8 mm×30 cm) (Tosoh-biosciences, Germany) and was eluted in 150 mmol/l sodium phosphate (pH 7) at the flow rate of 0.25 ml/min. The elution of RBD species was monitored by detecting the absorbance at 280 nm.

### CD studies

The far- and near-UV CD spectra were acquired in 0.1 mm and 10 mm path length cuvettes, respectively, at room temperature using a Jasco J-1500 spectropolarimeter equipped with the Peltier Jasco PTC-510 temperature controller and a mini-Jasco MCB-100 water circulation bath (Japan). An aqueous solution of recrystallized D-10-camphorsulfonic acid was used for routinely calibration of the instrument and all spectra obtained were the mean of five independent scans for far-UV CD and three for near-UV CD. The protein concentration was 4.13 μmol/l for mRBD and 2.07 μmol/l for dRBD in milliQ water for far-UV CD and 41.35 μmol/l for mRBD and 20.67 μmol/l for dRBD in PBS for near-UV CD. The baseline was corrected in all experiments using milliQ water for far-UV CD and PBS for near-UV CD. Collected data was analyzed with Spectral Manager II software prior to processing with Origin-v15.0 program (*Origin Lab Corporation, USA*), using a binomial smoothing through 20 neighbours. Data were expressed as mean residue molar ellipticity in deg.cm^2^.dmol^−1^. The secondary structure content of the proteins was estimated by the deconvolution of far-UV CD spectra according to SELCON III algorithm on the BeStSel Internet server (http://bestsel.elte.hu/index.php) [26].

### Fluorescence studies

Fluorescence spectra were recorded on a Jasco-1500 spectropolarimeter (Jasco Corporation, Japan) equipped with a Jasco FMO 522 fluorescence monochromator (Jasco FDT-538 fluorescence detector), Jasco PTC-510 Peltier temperature controller and Jasco MCB-100 water circulation mini-bath. Intrinsic protein fluorescence emission spectra were recorded using 10 mm path length quartz cuvettes from 307–410 nm, after excitation at 295 nm, to selectively obtain fluorescence spectra derived from Trp residues [20]. A bandwidth of 5 and 10 nm was used for excitation and emission slits, respectively. A protein concentration of 4.13 μmol/l for mRBD and 2.07 μmol/l for dRBD in PBS was used, and the baseline was corrected in all experiments using PBS.

### RBD stress stability

In this study, one batch of mRBD and one batch of dRBD were evaluated. To define the temperature at which the study would be carried out, it was taken into account that the storage temperature used for these products is – 20 °C, additionally the study of storage time of the purified RBD that was carried out at 2–8 °C. The temperature of 37 °C was then chosen to study the degradation products of the molecules in both their monomeric and dimeric forms. For this reason, the samples were incubated at 37 °C for 30 days [27] and measurements were made every 7 days. For time zero, the samples not subjected to said stress were used.

### ELISA

ELISA was performed to detect the binding of the SARS-CoV-2 RBD protein to the ACE2 receptor.

Microtiter plates were pre-coated with 10,000 µl of ACE2-hFc (CIM, Cuba) (5 μg/ml) in phosphate buffered saline (PBS) and incubated overnight at 4 °C. Plates were blocked with 200 µl/well of 4% of skim milk in PBS-Tween 0.05% (PBST) during 1 h, at 20–25 °C. Next, the blocking solution was discarded and 100 µl/well of samples was applied and incubated for 1 h at 20–25 °C. The bound protein was detected using 100 µl/well of RBD specific MAb CBSSRBD-S1 (CIGB, Cuba) (5 µg/ml in assay buffer) for 1 h at 20–25 °C. Next, 100µL/well of a peroxidase conjugated anti-mouse IgG antibody (Sigma, A2554) was added and incubated for 1 h at 20–25 °C. Then, the o-phenylendiamine dihydrochloride (OPD) peroxidase substrate (Sigma) was added and plates were light-protection incubated for 20 min at 20–25 °C. The reaction was stopped using 50 µl H_2_SO_4_ (Panreac, Spain). The optical density (OD) at 490 nm was measured using a ELISA microplate reader (Diareader, Germany). All incubations were followed by three washing steps with PBST.

## Data Availability

All data generated or analyzed during this study are included in this published article and supplementary information files.

## Abbreviations

ACE2: Angiotensin-converting enzyme 2
CD: Circular dichroism
COVID-19: Coronavirus disease 2019
dRBD: Dimeric receptor binding domain
ELISA: Enzyme-linked immunosorbent assay
FS: Fluorescence spectroscopy
IMAC: Immobilized metal affinity chromatography
GF-HPLC: Gel filtration high performace liquid chromatography
mRBD: Monomeric receptor binding domain
SARS-CoV-2: Severe acute respiratory syndrome coronavirus type 2
SDS-PAGE: Sodium dodecyl sulfate polyacrylamide gel electrophoresis
