# Correction: Conformational characterization of the mammalian-expressed SARS-CoV-2 recombinant receptor binding domain, a COVID-19 vaccine

**DOI:** 10.1186/s40659-024-00514-0

**Published:** 2024-06-01

**Authors:** Leina Moro-Pérez, Tammy Boggiano-Ayo, Sum Lai Lozada-Chang, Olga Lidia Fernández-Saiz, Beatriz Perez-Masson, Kathya Rashida de la Luz, Jose Alberto Gómez-Pérez

**Affiliations:** 1 Bioprocess R&D Department, Center of Molecular Immunology, 216 Street and 15 Avenue, Atabey, Playa, P.O. Box 16040, 11600 Havana Cuba; 2 Center of Molecular Immunology, Immunology and Immunobiology Direction, 216 Street and 15 avenue, Atabey, Playa, Havana City, P.O. Box 16040, 11600 Cuba

**Correction: Biological Research (2023) 56: 22**

10.1186/s40659-023-00434-5

In this article the author Beatriz Perez-Masson was omitted from the author group. Beatriz Perez-Masson has been added to the author group above and the original article has been corrected.

